# Improving the Assessment of Memory and Cognitive Side Effects Post Electroconvulsive Therapy

**DOI:** 10.1192/bjo.2024.370

**Published:** 2024-08-01

**Authors:** Amy-May Garvey, John Brady

**Affiliations:** WHSCT, Omagh, United Kingdom

## Abstract

**Aims:**

An April 2022 Electroconvulsive Therapy Accreditation Services (ECTAS) review of electroconvulsive therapy (ECT) services in the Southern Sector of the Western Health and Social Care Trust highlighted that the follow up of service users' memory and cognitive side effects post-ECT needed to be improved to deliver safer and more effective care. The aim of this MDT quality improvement project was to transform the follow-up process from a baseline of 13% of service users receiving memory assessment 1–2 months post ECT to 100% of service users receiving memory assessment 1–2 months post ECT over a 16 month period.

**Methods:**

In June 2022, an MDT working group was established with key stakeholders from inpatient and community mental health services. Using driver diagrams, opportunities for improvement were collectively identified and innovative ideas proposed to overcome these barriers. The primary drivers for change were communication, resources, and education. Systems were established and PDSA cycles used to review our data and decide whether we needed to make a further change. 17 service users received ECT and were followed up within the 16 month period. Our third change brought about the most significant and sustained improvement to the process; establish ECT champions within community teams. The ECT champion's role was to improve communication between inpatient and community teams in regards to service users needing memory follow up post ECT.

**Results:**

The introduction of three ECT champions within the community teams significantly improved communication between the inpatient and outpatient teams resulting in an improvement in the standard of care to our service users. Initial figures show 100% of service users having memory assessment follow up at 1–2 months post ECT in July 2023, October 2023 & December 2023. No service users required follow-up within the service in August/September/November 2023. Performance monitoring is ongoing as part of the service's governance meeting.

**Conclusion:**

In conclusion, by improving communication, utilising resources more effectively and educating through ECT champions, the percentage service users receiving memory assessments at 1–2 months follow up post ECT achieved ECTAS standard of 100%. This will benefit our service users by enabling us to identify those who need further input. Looking into the future, we need to undertake a clinical audit to assess for a sustained improvement and ensure that no unintended consequences have been introduced from this QIP. We have shared our learning within the wider trust and plan to spread and scale our changes across a wider area.

